# Supplementary material for: Mapping the evolution of fertility support policies in China: A content and instrumental analysis
Source: PLoS One. 2025 Oct 9;20(10):e0332137. doi: 10.1371/journal.pone.0332137 (PMC12510515; doi:10.1371/journal.pone.0332137)
Supplement: S1 Appendix — (ZIP) [file pone.0332137.s001.zip › S1 Appendix. 226 original policy documents/211-第十届全国人民代表大会第四次会议关于2005年国民经济和社会发展计划执行情况与2006年国民经济和社会发展计划的决议(FBM-CLI-1-75581).doc]

第十届全国人民代表大会第四次会议关于2005年国民经济和社会发展计划执行情况与2006年国民经济和社会发展计划的决议

**发布部门：** [全国人民代表大会](https://www.pkulaw.com/law/chl?Aggs.IssueDepartment=101)

**发布日期：**2006.03.14

**实施日期：**2006.03.14

**时效性：** [现行有效](https://www.pkulaw.com/law/chl?Aggs.TimelinessDic=01)

**效力级别：** [工作文件](https://www.pkulaw.com/law/chl?Aggs.EffectivenessDic=XA0105)

**法规类别：** [国家计划](https://www.pkulaw.com/law/chl?Aggs.Category=02404) [人大议事](https://www.pkulaw.com/law/chl?Aggs.Category=00106)

第十届全国人民代表大会第四次会议关于2005年国民经济和社会发展计划执行情况与2006年国民经济和社会发展计划的决议
(2006年3月14日第十届全国人民代表大会第四次会议通过)

　　第十届全国人民代表大会第四次会议审查了国务院提出的《关于2005年国民经济和社会发展计划执行情况与2006年国民经济和社会发展计划草案的报告》及2006年国民经济和社会发展计划草案，同意全国人民代表大会财政经济委员会的审查结果报告。会议决定，批准《关于2005年国民经济和社会发展计划执行情况与2006年国民经济和社会发展计划草案的报告》，批准2006年国民经济和社会发展计划。

关于2005年国民经济和社会发展计划执行情况
与2006年国民经济和社会发展计划草案的报告
--2006年3月5日在第十届全国人民代表大会第四次会议上
国家发展和改革委员会

各位代表：
　　受国务院委托，现将2005年国民经济和社会发展计划执行情况与2006年国民经济和社会发展计划草案提请十届全国人大四次会议审议，并请全国政协各位委员提出意见。
　　**一、**2005年国民经济和社会发展计划执行情况
　　2005年，全国各族人民在中国共产党的领导下，树立和落实科学发展观，加强和改善宏观调控，团结奋进，锐意进取，国民经济呈现增长比较快、效益比较好、价格比较稳的良好局面，完成了十届全国人大三次会议审议通过的国民经济和社会发展计划。
　　（一）经济继续保持平稳较快增长。经济运行的稳定性有所提高。全年国内生产总值达到182321亿元，增长9.9%，四个季度分别增长9.9%、10.1%、9.8%和9.9%。规模以上工业增加值全年增长16.4%，四个季度分别增长16.2%、16.5%、16.2%和16.4%。（见图表一）
　　经济发展的协调性有所改善。呈现增速较高、物价平稳的格局，全年居民消费价格总水平涨幅为1.8%。效益与速度同步提高，全年财政收入增长19.8%，增收5231.5亿元，规模以上工业企业实现利润增长22.6%。经济增长支撑条件绷得过紧的状况有所缓解，煤炭产量21.9亿吨，增长9.9%；发电量2.47万亿千瓦小时，增长12.3%，迎峰度夏期间国家电网系统拉限电条次和损失电量同比分别减少61%和66%；重点物资运输得到较好保障。（见图表二）
　　（二）产业结构调整取得新进展。农业得到进一步加强。中央财政用于“三农”的支出达到2975亿元，比上年增加349亿元。粮食播种面积比上年增加267万公顷，粮食产量达4840亿公斤，增加145.4亿公斤。农业优质化、区域化、产业化和标准化程度继续提高，全国优质专用小麦种植面积占全部小麦种植面积的比重达48%，优质稻种植面积比重为64%，分别提高4个和3个百分点。畜牧水产业发展势头良好。（见图表三）
　　工业结构升级步伐加快。高技术产业快速发展，规模以上工业中，高技术产业增加值增长19.8%。一批重大科技专项和产业化专项顺利实施。重大关键技术和装备国产化取得新进展。重型燃气轮机、城市轨道交通运输装备、大型水电和抽水蓄能机组的引进消化吸收工作顺利推进，百万吨级乙烯装备国产化依托工程开始启动，建成750千伏交流输变电示范工程。企业兼并重组步伐加快，淘汰了一批高耗能、高污染和不符合安全生产条件的落后生产能力，部分过度扩张行业投资增幅继续回落。（见图表四）
　　基础设施建设继续加强。西电东送、南水北调等重点工程建设进展顺利。青藏铁路提前一年全线铺通。县际公路改造工程基本完成。新建公路13万公里，其中高速公路6457公里。新建铁路投产里程1203公里，新增电气化铁路863公里，新增铁路复线486公里。
　　服务业继续稳定发展。政府和企业信息化应用水平进一步提高。邮电通信、现代物流、社区服务、旅游、会展等新兴服务业发展加快，商贸、餐饮等传统服务业保持良好发展势头。
　　（三）区域发展总体战略积极推进。西部大开发进展顺利，新开工重点工程10项，投资总规模1360亿元。遂宁-重庆-怀化铁路建成投产，“五纵七横”国道主干线西部路段全部开工，重庆、西宁、满洲里等10个西部机场新建、改扩建工程先后完工，百色、紫坪铺等水利工程相继下闸蓄水。东北地区等老工业基地振兴战略扎实推进。以重大装备国产化和提升企业自主创新能力为重点，支持石化、钢铁、重大装备、造船、汽车和零部件等优势产业发展壮大。资源型城市经济转型试点取得阶段性成果，棚户区改造开始实施。研究制定了促进中部崛起的政策措施，中部地区农业基础进一步巩固，优势产业发展壮大，城市群的辐射带动作用增强。东部地区继续保持率先发展的良好态势，经济结构调整、增长方式转变取得新的成效。
　　（四）经济体制改革实现新突破。农村综合改革试点积极推进，取消了牧业税，全部免征农业税的省份扩大到28个。国有企业改革继续深化，国有资产监管力度加大。铁路、民航、烟草等行业管理体制改革迈出新步伐，邮政体制改革开始启动。制定并实施了鼓励支持和引导非公有制经济发展的政策措施。人民币汇率形成机制改革顺利实施。上市公司股权分置改革稳步推进。国有商业银行股份制改革迈出重要步伐。深化农村信用社改革试点全面展开。调整完善了出口退税机制。东北老工业基地增值税转型改革试点进展顺利。投资体制改革继续推进，企业投资项目核准制和备案制全面实施。天然气出厂价格改革方案顺利出台，煤电、煤热价格联动机制初步建立，水价改革稳步实施。
　　（五）对外开放迈出新步伐。外贸进出口总额14221亿美元，增长23.2%。其中，出口7620亿美元，增长28.4%；进口6601亿美元，增长17.6%。机电产品和高新技术产品出口分别增长32%和31.8%。服务业对外开放进一步扩大。控制高耗能、高污染和资源性产品出口取得成效。利用外资继续保持较大规模，水平进一步提高，全年实际利用外商直接投资603亿美元。年末外汇储备8189亿美元。企业“走出去”取得积极进展，全年对外直接投资（非金融部分）达69亿美元。（见图表五）
　　（六）资源节约和环境保护取得新成效。节能、节水、节材、节地、资源综合利用和发展循环经济工作力度加大。在重点行业、重点领域、产业园区和部分地区启动了国家第一批循环经济试点。对部分高耗电行业实行了差别电价政策，对部分高耗水行业制定了取水定额国家标准，对部分产品发布了强制性能效标准。万元国内生产总值用水量下降8.7%。节能建筑推广、墙体材料革新及木材节约和代用取得新进展。全社会资源忧患意识与节约意识进一步增强。
　　“三河三湖”、三峡库区、南水北调工程沿线等重点流域区域水污染防治工作步伐加快，城镇污水、工业废水、危险废物、火电厂二氧化硫污染治理进一步加强。城市污水处理率和工业固体废物综合利用率继续提高。部分空气污染严重城市的空气质量有所改善。退耕还林完成111.1万公顷，荒山荒地造林及封山育林266.7万公顷，退牧还草666.7万公顷。天然林资源保护、京津风沙源治理等重点生态建设工程进展顺利。“三化”草地治理、水土流失治理和矿山生态恢复面积分别为838万公顷、420万公顷和7.2万公顷。野生动植物资源保护得到加强。（见图表六）
　　（七）科技、教育、文化、卫生等社会事业全面发展。制定并公布实施《国家中长期科学和技术发展规划纲要（2006-2020年）》。国家创新体系、基础研究和科技基础设施建设进一步加强。神舟六号载人航天飞行取得圆满成功。龙芯2号高性能通用计算机芯片、禽用禽流感基因工程灭活疫苗研制成功。抗虫棉推广面积达300万公顷。全年共签定技术合同26.5万项；技术合同成交额1510亿元，比上年增长13.2%。
　　全国义务教育人口覆盖率超过95%。西部地区“两基”攻坚计划取得新进展。新建、改扩建农村寄宿制学校2400多所，为16万个农村中小学和教学点配备了远程教育设施。中央和地方财政安排专项资金70多亿元，对592个重点贫困县1700万名贫困家庭学生免除学杂费、免费提供教科书和补助寄宿生生活费，还为中西部地区1700多万名贫困家庭学生免费提供教科书。中等职业学校和县级职教中心的校舍和实训设施建设步伐加快。高等教育“211工程”、“985工程”稳步推进。（见图表七）
　　公共文化体系建设进一步加强。重大文化项目建设和自然文化遗产保护力度加大，基本实现县县有文化馆和图书馆。广播电视村村通工程取得新进展，新增覆盖人口2700万。《全国红色旅游发展规划纲要》开始实施。全民健身运动深入开展，奥运场馆建设进展顺利。
　　疾病预防控制体系基本建成。医疗救治体系建设进展顺利。农村卫生服务体系建设继续推进，以中西部乡镇卫生院为重点的农村卫生基础设施建设得到加强。新型农村合作医疗制度试点范围扩大到671个县，有1.77亿农民参加合作医疗。城市社区卫生服务发展加快。高致病性禽流感疫情防控取得初步成效。农村计划生育服务体系建设开始实施，全国人口自然增长率5.89‰。
　　（八）人民生活进一步改善。城镇居民人均可支配收入和农村居民人均纯收入分别实际增长9.6%和6.2%。社会消费品零售总额增长12.9%。城镇新增就业970万人，城镇登记失业率4.2%。社会保障覆盖范围继续扩大，“两个确保”进一步巩固，城乡困难群众基本生活得到保障。全国绝大多数地方已基本实现国有企业下岗职工基本生活保障向失业保险并轨，东北三省完善城镇社会保障体系试点顺利实施。（见图表八、图表九 ）
　　经济社会发展在取得成绩的同时，也面临不少困难和挑战。制约我国经济健康发展的长期性、深层次问题仍然是：自主创新能力弱，经济结构不合理；增长方式粗放，资源环境约束加剧；城乡经济二元结构矛盾突出，经济社会发展不协调；体制机制不完善，法制环境不健全等。当前比较突出的是：（1）粮食增产和农民增收难度加大。农业基础薄弱的状况没有根本改变，依靠扩大种植面积增加粮食产量的余地不大。农业总体效益低，粮食价格下行压力较大，能源、原材料价格上涨导致农资生产成本提高，农民增加务工收入的制约因素较多。（2）固定资产投资增幅仍然偏高。2005年全社会固定资产投资增长25.7%，增速虽比上年有所回落，但仍在高位运行。在建规模偏大，新开工项目偏多，有些行业投资增长过快，投资结构不合理，投资反弹压力较大。（3）部分行业产能过剩的不良后果开始显现。导致相关产品价格下跌，库存上升，企业利润增幅下降、亏损增加，金融潜在风险加大。（4）涉及群众切身利益的不少问题还没有得到很好解决。就业再就业压力加大，社会保障体系不健全，部分社会成员之间收入差距仍有继续拉大趋势，教育乱收费、群众看病难和看病贵问题没有明显改观。在环境保护、企业改制、土地征用、房屋拆迁、库区移民等方面还存在不少群众反映强烈的问题。高致病性禽流感和重大动植物疫病防控任务较重。（5）安全生产形势相当严峻。煤矿、交通等重特大事故频繁发生，给人民群众生命财产造成严重损失。对上述问题，要高度重视，积极采取措施，认真加以解决。

　　**二、**2006年经济社会发展的总体要求和主要目标
　　今年是“十一五”的开局之年，是我们在新的历史起点上继续前进的一年。做好今年的各项工作，对于巩固和发展经济平稳较快发展的好势头，顺利实施“十一五”规划至关重要。2006年经济社会发展工作，要以邓小平理论和“三个代表”重要思想为指导，认真贯彻党的十六大和十六届五中全会精神，坚持以科学发展观统领经济社会发展全局，保持宏观经济政策的连续性和稳定性，着力加快改革开放，着力增强自主创新能力，着力推进经济结构调整和经济增长方式转变，着力提高经济增长的质量和效益，坚持把解决涉及人民群众切身利益问题放在突出位置，正确处理改革发展稳定的关系，全面加强社会主义经济建设、政治建设、文化建设与和谐社会建设，为“十一五”开好局、起好步。
　　按照上述要求，做好今年经济社会发展工作，必须坚持“稳定政策、适度微调，把握大局、抓好重点，统筹兼顾、关注民生，立足当前、着眼长远”的原则。综合考虑经济社会发展的需要和可能以及上年实际执行情况，并与“十一五”规划纲要（草案）相衔接，2006年经济社会发展的主要目标是：
　　--国内生产总值增长8%左右。主要考虑是：我国资源环境压力加大，经济运行总体上仍绷得较紧，发展中还存在一些不稳定、不确定因素，增长速度不宜定得过高；从有利于增强综合国力、扩大就业和缓解各种社会矛盾的需要，以及近几年增长速度较快的情况出发，增长速度又不宜定得过低。8%左右是全国经济增长的预期目标，各地要从实际出发，合理、适度地确定本地增长速度，防止盲目追求和攀比速度，切实把主要精力放到提高经济增长质量和效益上来。
　　--城镇新增就业900万人，城镇登记失业率控制在4.6%。今年就业压力仍然较大，推进企业改革和结构调整，会在短期内影响就业岗位的增加。按照经济增长与提供就业岗位的关系测算，通过完善和落实积极的就业政策，这一目标是可以实现的，但必须付出艰苦努力。
　　--居民消费价格总水平上涨3%。这一指标比上年实际略高一些，主要考虑是：国际油价高位运行会对国内相关产品价格产生一定影响；理顺资源性产品价格关系，疏导价格矛盾，调整部分服务类价格，也会在一定程度上推动价格总水平上升。
　　--外贸进出口总额增长15%。要继续保持外贸进出口稳定增长。鉴于目前出口规模已经较大，且相当多的出口商品附加值较低，外贸增长目标不宜定得过高，以有利于引导各方面着力转变外贸增长方式，加快调整进出口商品结构，促进国际收支基本平衡。
　　--单位国内生产总值能耗降低4%左右。我国目前仍处在资源消费强度较高的时期，强化节能降耗措施见效需要一个过程，实现这一目标的难度较大。但我国能源效率低下的状况已难以为继，同时节能降耗的潜力很大，只要加大调整经济结构力度、加快转变粗放型增长方式，这一指标必须也能够实现。
　　--城乡居民收入稳定增长。城镇居民人均可支配收入和农村居民人均纯收入分别实际增长6%和5%。主要考虑是：随着经济较快增长，企业效益继续提高，国家促进农民增收、加强社会保障和扶贫工作等各项措施逐步落实，城乡居民收入将持续增加；但提高居民特别是农民收入水平也面临许多困难和制约因素。在城乡居民收入稳定增长的基础上，消费需求保持平稳较快增长，全社会消费品零售总额预期增长12%。
　　--科技、教育等各项社会事业加快发展。研究与试验发展经费支出占国内生产总值的比重力争达到1.45%。高中阶段教育毛入学率达到57%，普通高校计划招生530万人，研究生40万人。公共文化服务体系建设进一步加强。新型农村合作医疗试点范围扩大到全国40%左右的县（市、区）。人口自然增长率控制在7.5‰以内。
　　--生态环境状况继续改善。主要污染物排放总量减少2%。工业固体废物综合利用率提高到56.2%。城市污水处理率达到50%。

　　**三、**2006年经济社会发展的主要任务和措施
　　实现今年国民经济和社会发展目标，要统筹安排、突出重点，务必保持经济平稳较快增长的良好势头，务必在推进社会主义新农村建设上有良好开端，务必在增强自主创新能力、推进结构调整上取得明显进展，务必在节能降耗、转变增长方式上取得显著成效，务必在重点领域和关键环节的改革上取得新突破，务必在构建社会主义和谐社会上迈出新步伐。为此，要着力抓好以下十方面工作。
　　（一）稳定宏观经济政策，保持经济平稳较快发展。继续实施稳健的财政政策和稳健的货币政策。（1）适当减少中央财政赤字，继续调整财政支出结构。2006年，全国财政预算收入35423亿元，支出38373亿元，中央财政赤字拟安排2950亿元，比上年预算减少50亿元，财政赤字占国内生产总值的比重进一步降低。大力开展增收节支，依法加强收入征管，严格控制一般性支出增长，保障重点支出需要。（2）缩减长期建设国债发行规模，合理确定投资方向。2006年拟发行长期建设国债600亿元，比上年减少200亿元；中央预算内经常性建设资金554亿元，增加100亿元。按照突出重点、压缩一般的原则，进一步调整国债资金和预算内投资结构，优先安排重点续建项目，适当开工建设事关“十一五”发展全局的重大项目。中央政府投资的主要方向是：改善农村生产生活条件，推进社会主义新农村建设；促进基础教育、公共卫生等社会事业发展；支持西部大开发等区域协调发展；加强环境保护与生态建设、水利、交通、能源等重大基础设施建设，以及涉及人民群众生命财产安全和切身利益的建设项目等。加强稽察监管，提高投资效益。（3）保持货币信贷适度增长，着力优化信贷结构。2006年，广义货币供应量（M2）和狭义货币供应量（M1）分别预期增长16%和14%，新增人民币贷款2.5万亿元。合理控制中长期贷款规模。积极引导金融机构增加对有效益、有市场企业的流动资金贷款，着力解决农户、中小企业等贷款难问题，加大对经济社会发展薄弱环节和西部大开发、东北地区等老工业基地调整改造、中部崛起的信贷支持。发展直接融资，扩大企业债券发行规模。继续推进利率市场化改革，健全货币政策传导机制。完善有管理的浮动汇率制度，保持人民币汇率在合理、均衡水平上的基本稳定。进一步改进外汇管理，优化储备结构，加强对跨境资本流动的监控。（4）保持价格总水平基本稳定。继续加强对价格总水平和粮食、棉花、石油、煤炭、钢材、商品房等重要商品和服务价格走势的监测分析。做好市场价格巡查工作。
　　加强固定资产投资调控。2006年全社会固定资产投资总规模预期增长18%。继续严把土地、信贷两个闸门，从严控制新开工项目，抑制固定资产投资过快增长，防止投资反弹。（1）坚持实行最严格的土地管理制度，从紧控制农用地特别是耕地转为建设用地。2006年，农用地转为建设用地的规模控制在26.67万公顷。（2）坚持区别对待、有保有压的原则，严格控制过度扩张行业的土地和资金供应，保障国家重点建设项目、有利于结构调整重大项目的建设用地和资金需要。（3）加强城市规划，合理控制拆迁规模，规范城建项目打捆贷款。巩固清理整顿开发区成果，进一步完善政策。（4）着力调整住房供应结构，增加普通商品房和经济适用房供给，健全商品房与经济适用房、廉租住房相结合的城镇住房供应体系，控制高档房地产开发，基本完成建设领域清理拖欠工程款任务，引导建筑业与房地产市场健康发展。
　　着力扩大消费需求。（1）努力增加城乡居民尤其是中低收入者的收入，适当提高企业离退休人员基本养老金标准、残疾军人等各类优抚对象抚恤补助标准、城市居民最低生活保障补助标准。合理调整并严格执行企业最低工资标准，制定和推行最低小时工资标准。根据《[中华人民共和国公务员法](https://www.pkulaw.com/chl/823ca97b7f55d18cbdfb.html)》，改革公务员工资制度。推进事业单位收入分配制度改革。（2）改善消费心理预期，通过加快完善社会保障体系和解决教育、医疗、住房等领域的突出问题，稳定居民支出预期，扩大即期消费。（3）积极开拓农村市场，加快农村商业流通体系建设，加大对农村商业网点连锁经营、农资物流配送等项目的支持力度，继续实施“万村千乡”市场工程。发展城市社区商业和服务业。（4）培育新的消费热点，扩大文化、健身、旅游等服务性消费，规范和发展住房、汽车交易市场。（5）改善消费环境，合理调整现行消费税，加快个人征信体系建设，完善消费信贷政策。积极发展电子商务。继续开展教育、医药价格收费专项检查，严厉查处商业欺诈行为，切实保障消费者合法权益。
　　促进煤电油运供需有效衔接。在保证安全的前提下，稳定煤炭生产和供应，推进大型煤炭基地建设。加快水电、核电、可再生能源等清洁能源建设，优化发展火电，完善电网结构。努力增加石油天然气供给，推进煤炭液化示范、煤层气开发利用工程，加快建立石油储备体系。继续加强煤运通道、下水港口和其他重大交通基础设施建设。建立电力需求侧管理的长效机制。搞好煤电油运综合协调，着力解决与群众生活密切相关的供水、供气、供电、供暖和供油等问题。
　　（二）推进社会主义新农村建设，促进农业发展和农民增收。坚持“多予少取放活”的方针，加快建立以工促农、以城带乡的长效机制。（1）稳定发展粮食生产。继续加强农业综合生产能力建设，努力使粮食总产量保持在上年水平。搞好土地管理和基本农田保护，稳定粮食播种面积。加强大型商品粮基地建设，继续实施优质粮食产业工程、种子工程和植保工程。增加对产粮大县和财政困难县的转移支付。继续对短缺的重点粮食品种在主产区实行最低收购价政策，稳定粮食市场价格。健全中央和省级粮食储备调节制度和调控机制，进一步充实地方储备。（2）发展现代农业。按照“高产、优质、高效、生态、安全”的要求，积极推进优势农产品的区域化布局、专业化生产和产业化经营。加快农业科技进步，大力推广农业先进适用技术。建设新疆优质棉基地和长江流域“双低”油菜产业带。继续实施畜禽水产良种工程，支持有条件的地区发展养殖小区。加快实施动物防疫体系建设规划，落实好扶持家禽业发展的各项措施。（3）着力改善农村生产生活条件。把国家对基础设施建设投入的重点放在农村，继续加强以小型水利建设为重点的农田基本建设，支持大型灌区节水改造和中部地区排涝泵站改造，突出抓好农村“水、气、路、电”等基础设施和人居环境建设。2006年，用于农村饮水安全工程的中央投资拟比上年增加1倍，再解决2000万高氟、高砷、苦咸水、污染水和季节性缺水地区农村人口的饮用水安全问题。用于农村沼气建设的中央投资拟比上年增加1.5倍，新增农村沼气用户250万户，带动农户改圈、改厕、改厨。启动“十一五”农村公路改造工程，加强东部、中部地区的通村沥青（水泥）路和西部地区的通乡沥青（水泥）路建设。加快无电村电力建设，实施部分省份农网完善工程。（4）加快发展农村公共事业。今年要在西部地区全部免除农村义务教育阶段学生学杂费。继续对农村义务教育阶段贫困家庭学生免费提供教科书并补助寄宿生生活费。提高农村义务教育阶段中小学公用经费保障水平，建立和完善农村中小学校舍维修改造投入机制、教师工资经费保障机制。基本完成农村寄宿制学校改造任务和农村中小学现代远程教育工程。实施《农村卫生服务体系建设与发展规划》，以中西部为重点继续推进农村卫生基础设施建设，再改建和新建4200个乡镇卫生院，达到规划目标的68%。扩大新型农村卫生合作医疗制度的试点范围，推行农村医疗救助制度。启动20户以上通电自然村的广播电视村村通工程，继续实施农村电影放映工程，推动乡镇文化站建设。（5）多渠道增加农民收入。稳步推进农业产业化经营，积极发展贸工农一体化和农副产品加工龙头企业及各类专业合作经济组织，挖掘农业内部增收潜力。增加对农民的种粮直接补贴、良种补贴、农机具补贴，对种粮农民逐步实行化肥、柴油等农资直接补贴制度。加强化肥价格和涉农收费的监管，抑制农业生产资料价格上涨，切实减轻农民负担。强化农民工就业培训，继续清理对农民工的歧视性政策，落实农民工工资支付措施，促进农村劳动力向城镇和非农产业转移就业。发展壮大县域经济。
　　（三）大力增强自主创新能力，扎实推进创新型国家建设。认真实施《国家中长期科学和技术发展规划纲要（2006-2020年）》，大力推进原始创新、集成创新和引进消化吸收再创新。（1）加强重大科技攻关。在条件成熟的基础上，启动实施重大新药创制、转基因生物新品种培育等若干重大科技专项。实施蛋白质研究、纳米研究等重大科学研究计划。在能源、资源、信息、装备等领域，集中力量组织一批重大产业核心技术攻关。加强生态环境、人口与健康、公共安全等领域的公益性技术研究。继续繁荣和发展哲学社会科学。（2）加快国家创新体系建设。强化企业在自主创新中的主体地位，培育3000家左右行业骨干企业技术中心。新建煤矿瓦斯治理、新型疫苗、船舶制造等一批国家工程中心。启动建设下一代互联网应用、生物冶金等若干国家工程实验室。积极推动海洋科学综合考察船、新一代大型天文望远镜等九大科技基础设施建设。建设中科院知识创新三期工程。调整和完善国家科技计划体系，优化科技资源配置，促进科技成果转化。继续推进技术开发类院所企业化转制和社会公益类科研机构改革。完善科技评审评估和成果评价奖励等制度。大力培育和发展科技中介服务机构。加强科学普及工作，实施全民科学素质行动计划。（3）进一步细化鼓励自主创新的财税、金融、政府采购等政策措施，鼓励企业加大研发投入。支持使用、制造和设计单位联合招标引进重大技术。建立财政性科技投入稳定增长机制。设立创业风险投资引导基金。对选用国产首台、首套装备给予政策扶持。加强对重大技术和装备引进的管理，防止盲目重复引进。支持中小企业提升自主创新能力，鼓励外资企业研发活动本地化。加大对知识产权的保护力度。（4）加强科技人才队伍建设。建立健全人才评价机制。依托国家重点项目、重点学科和重点科研基地，造就一批科技领军人才、学科带头人和战略科学家。
　　（四）加快推进产业结构优化升级，大力提升产业技术水平和整体竞争力。（1）着力推进部分产能过剩行业的结构调整。综合运用经济、法律和必要的行政手段，充分发挥市场机制作用，促进优胜劣汰。加强产业政策引导、信贷政策调节、财政政策支持，继续完善行业规划，贯彻落实《促进产业结构调整暂行规定》，提高并严格执行环保、安全、技术、土地和资源综合利用等市场准入标准，严格控制新增产能。继续清理整顿在建和拟建项目，对不符合市场准入条件的项目，依法停止建设。依法关闭一批破坏资源、污染环境和不具备安全生产条件的企业。围绕提升技术水平、改善品种、保护环境、降低消耗、保障安全等，对传统产业实施改造提高。按照市场原则，鼓励钢铁、水泥、电解铝、煤炭等行业兼并重组，支持优势企业做强做大，提高整体技术水平和行业集中度。（2）加快发展高技术产业。改善高技术企业的信贷服务和融资环境。重点支持集成电路、软件和新型元器件等核心产业，培育数字化音视频、下一代互联网、新一代移动通信、高性能计算机及网络设备等信息产业群。积极发展生物产业。加快推进广播电视直播卫星、新支线飞机工程。坚持以信息化带动工业化，广泛应用高新技术和先进适用技术改造提升传统产业。（3）大力振兴装备制造业。完善支持重大装备发展的政策和配套措施。依托重点工程，继续推进燃气轮机、超临界和超超临界燃煤发电机组、百万千瓦核电机组、高速列车、大型煤化工成套设备等16个重点领域的重大装备国产化，带动基础产品和零部件的发展。（4）促进服务业全面发展。选择若干城市开展服务业体制改革与创新试点。加快发展交通运输、金融、信息、现代物流、各种中介服务组织等生产性服务业和社区服务、旅游等新兴消费性服务业。提高商贸、餐饮、邮政等传统服务业的水平和质量。
　　（五）加强资源节约和环境保护，促进经济增长方式转变。（1）积极推进节能降耗。抓紧制定和完善各行业节能、节水、节材、节地标准，建立各地区和重点行业能耗目标考核体系和单位国内生产总值能耗公报制度。实施十大重点节能工程，突出抓好1000家高耗能企业的节能工作。扩大节能产品认证范围，推行强制性能效标准，加强重点用能产品能效标识管理。继续推进政府部门采购和使用节能产品。发展农业节水灌溉。支持高耗水行业节水技术改造，加快再生水利用设施建设，支持海水淡化和直接利用，加大节水设备和器具的推广力度。加强重点行业原材料消耗管理。鼓励开发高强度和耐腐蚀金属材料，促进木材节约和代用。强化节约和集约利用土地，建立和完善建设用地定额标准，发展节能省地型建筑。推动第二批城市限时禁用实心粘土砖。（2）大力发展循环经济。完善发展循环经济、促进资源节约和综合利用的财税、价格政策。组织实施国家循环经济试点。支持循环经济关键和共性技术研发及产业化示范。推进共伴生矿产资源和工业废物综合利用，做好再生资源回收利用。广泛开展宣传教育，倡导健康文明、节约资源的消费模式和生活方式。（3）搞好生态建设和环境保护。继续实施天然林资源保护、水土保持、京津风沙源治理、三江源自然保护区生态保护和建设、三峡库区周边绿化带建设等重点生态工程。抓紧建立生态补偿机制。加大“三河三湖”、环渤海、三峡库区、南水北调沿线等重点流域区域水污染防治力度。严格保护城镇饮用水源地水质。加强现有燃煤电厂烟气二氧化硫排放治理。做好城市大气污染综合防治工作。推行清洁生产。加强核安全和辐射环境的监管。认真履行《[联合国气候变化框架公约](https://www.pkulaw.com/eagn/a436a870449c3f51eed86ee7d60657cdbdfb.html)》。大力发展环保产业。
　　（六）充分发挥比较优势，推动区域协调发展。（1）着力促进西部地区重点地带、重点城市和重点产业开发，努力增强自身发展能力。新开工一批重大基础设施工程。巩固和发展退耕还林、退牧还草成果。继续加强长江、黄河上游水污染治理。加大矿产资源勘探开发力度，支持建设石油、稀土、钾磷肥等特色资源加工基地。进一步加强西部地区科技教育、人才开发和法制建设。加快建立长期稳定的西部开发资金渠道。（2）加强东北大型粮食基地建设，促进重点行业结构调整和国有企业改革重组，抓好资源型城市经济转型、采煤沉陷区治理和棚户区改造工作，实施以松花江、辽河流域为重点的生态环境整治工程。抓紧研究建立资源开发补偿机制、衰退产业援助机制。做好东北地区厂办大集体改革试点工作。认真落实扩大对内对外开放的政策措施。（3）出台并组织实施促进中部崛起的指导意见，在产业发展、重大基础设施建设、资金投入、推进改革开放等方面加大支持力度。重点加强现代农业特别是粮食主产区商品粮基地、能源重要原材料基地、现代装备制造和高技术产业基地以及综合交通运输体系建设。发展现代流通业。支持老工业城市加快企业改组改造和资源型城市转型。增强中心城市辐射功能。（4）东部地区要率先增强自主创新能力、国际竞争力和可持续发展能力，更加注重节约利用土地、水、能源、矿产资源，加快发展高技术产业和现代服务业。继续发挥经济特区、上海浦东新区的作用，推进天津滨海新区开发开放，支持海峡西岸和其他台商投资相对集中地区的经济发展。积极发展海洋经济。（5）进一步支持革命老区、少数民族地区、边疆地区和贫困地区的经济社会发展。加大对人口较少民族的扶持力度，推进兴边富民行动。健全发达地区帮扶欠发达地区发展的机制，继续做好区域合作对口支援工作。（6）开展主体功能区划研究，合理确定优化开发、重点开发、限制开发、禁止开发四类功能区的范围，根据各区域的主体功能定位，确定发展方向和配套政策。
　　（七）继续深化改革，营造科学发展的体制环境。加强对改革的总体指导、统筹协调和综合配套，力争在一些重要领域和关键环节继续取得新突破。（1）全面推进以深化乡镇机构、农村义务教育和县乡财政管理体制改革为主要内容的农村综合改革。规范农村土地流转政策，加快征地制度改革。深化粮棉流通体制改革。完善农村信用社体制，做好农业银行、农业发展银行改革和政策性农业保险试点工作，改进农村金融服务。（2）深化企业改革。以贯彻修订后的[公司法](https://www.pkulaw.com/chl/e54c465cca59c137bdfb.html)为契机，推进国有大型企业股份制改造，着力完善产权结构、法人治理结构和激励约束机制，加快形成一批拥有自主知识产权、知名品牌和国际竞争力的大公司、大企业集团。继续做好企业政策性关闭破产、分离办社会职能和主辅分离工作。规范国有企业改制和国有产权转让行为，防止国有资产流失，维护职工合法权益。加快建立国有资本经营预算制度，建立健全非经营性资产、自然资源资产等监管体制。进一步深化电信、电力、民航、邮政、烟草管理体制改革。推进铁路投融资体制改革，研究提出铁路体制改革方案。进一步引入市场机制，完善市场监管，深化供水、供气、供热等市政公用事业改革。积极推动集体企业改革和发展。完善鼓励支持和引导非公有制经济发展的配套政策措施，推动实施中小企业成长工程。（3）加快推进金融体制改革。继续做好国有商业银行股份制改革工作，坚持国家绝对控股，完善公司治理结构和内控机制。积极稳妥地推进资本市场改革和发展，着力提高上市公司质量，搞好证券公司的综合治理，继续开展股权分置改革和产业投资基金试点。深化保险业改革，推进政策性银行、邮政储蓄机构等其他金融机构改革。规范金融市场，强化金融监管，维护金融稳定和安全，防范和化解金融风险。（4）深化财税体制改革。健全公共财政体系，完善转移支付制度，实施政府收支分类改革，完善预算管理制度。积极推进增值税转型改革，调整和完善资源税，研究统一各类企业税收制度。（5）深化投资体制改革。进一步完善投资项目核准制和备案制。尽快制定中央预算内直接投资管理和中央政府投资项目决策责任追究等办法。健全投资宏观调控体系。（6）积极稳妥推进资源性产品价格改革。继续实行峰谷、丰枯分时电价制度，稳步推进电力竞价上网，实行鼓励可再生能源发展的电价机制。完善石油价格形成机制。调整天然气出厂价格。加大水资源费征收力度，合理调整城市供水和水利工程供水价格，全面开征污水处理费，在有条件的地方加快实施阶梯式水价和超计划超定额用水加价制度。建立规范的基准地价制度。价格改革要兼顾各方面利益，尤其是要采取措施，保证低收入群众基本生活不受影响。（7）继续推进行政管理体制改革。深入贯彻[行政许可法](https://www.pkulaw.com/chl/6cc71b5743a5ee2abdfb.html)，进一步转变政府职能，减少和规范行政许可。健全对涉及经济社会发展全局重大事项决策的协商和协调机制，完善专家论证、技术咨询、决策评估制度和公示、听证制度，提高决策的科学化、民主化水平。积极推进事业单位改革。
　　（八）积极实施互利共赢开放战略，进一步提高对外开放质量和水平。（1）加快转变外贸增长方式，优化进出口商品结构。大力实施科技兴贸战略。完善出口退税、金融支持和品牌认证等措施，提高出口产品的质量、档次和附加值。支持企业扩大具有自主知识产权、自主品牌的高新技术和机电产品出口。推动加工贸易转型升级，提高产业层次和加工深度。控制高耗能、高污染和资源性产品出口。着重增加先进技术、关键设备和国内短缺资源进口，努力改善贸易不平衡状况。做好加入世贸组织后过渡期工作。加强区域和多双边经贸合作。进一步落实内地与港澳更紧密经贸关系安排的各项措施。（2）继续积极利用外资，优化结构、提高质量。注重引导跨国公司向我国转移附加值高、技术含量高的加工制造环节、服务外包业务和建立技术研发机构。加强对外资投向的产业和区域引导，完善规范企业并购的相关政策，严格限制低水平、高消耗、高污染的外资项目进入，维护产业安全。（3）积极稳妥地实施“走出去”战略。继续支持和引导有条件的企业对外投资，完善境外投资的相关政策、法律法规以及促进和保障体系，健全协调和监管机制。
　　（九）坚持把教育放在优先发展的战略位置，促进各项社会事业全面进步。（1）优先发展教育事业。全面实施素质教育。普及和巩固九年义务教育，强化政府对义务教育的保障责任，改善农村办学条件，解决好城市低收入家庭和进城务工农民的子女上学问题。启动实施职业教育基础能力建设工程，专项支持县职教中心、中等职业学校和示范性高等职业院校建设。推进高中阶段教育协调发展。继续实施“211工程”和“985工程”，提高高等教育质量。积极发展民办教育。（2）完善公共卫生和基本医疗服务体系。全面完成医疗救治体系建设任务。调整和优化城市医疗资源布局，构建以社区为基础的新型城市医疗卫生服务体系。继续做好艾滋病、结核病、血吸虫病等重大疾病的防治及高致病性禽流感的防控工作。推动医疗卫生体制和药品生产流通体制改革。继续降低药品价格，整顿医疗器械市场价格秩序，加强医德医风建设，规范医疗服务中的诊疗和用药行为，抓紧解决群众看病难、看病贵问题。支持中医药事业发展。（3）大力发展文化、广播影视、新闻出版、旅游、体育等事业。深化文化体制改革。积极推进文化设施建设，加强重大自然文化遗产、民间文化保护。做好新闻出版工作。围绕全国重点红色旅游区和精品路线，加强经典景区和相关景区的基础设施建设。积极推进全民健身运动，提高竞技体育水平。继续抓好北京奥运会和上海世博会筹办工作。（4）稳定现行生育政策和低生育水平，提高出生人口素质。以中西部地区县级和中心乡计划生育服务站建设为重点，健全基层计划生育服务网络，完善流动人口计划生育管理和服务。扩大农村计划生育家庭奖励扶助制度的覆盖面和西部地区“少生快富工程”的实施范围。大力发展老龄事业。
　　（十）认真解决关系人民群众切身利益的问题，高度重视和维护社会稳定。以扩大就业、完善社会保障体系、理顺分配关系、维护社会稳定为着力点，扎实推进和谐社会建设。（1）继续实施积极的就业政策。进一步解决体制转轨遗留的下岗失业人员再就业问题和重组改制关闭破产企业的职工安置问题。大力发展服务业、劳动密集型产业和中小企业，拓宽就业渠道。做好新增劳动力特别是高校毕业生、退役军人和就业困难人员的就业指导和服务工作。大力开展职业培训和就业服务体系建设。完善对特殊困难群众的就业援助制度。鼓励自谋职业和自主创业，支持多种形式灵活就业。加强劳动者权益保护工作。（2）加快完善社会保障体系。切实保证各项社会保险待遇及时支付。继续扩大城镇社会保险覆盖面。完善城镇基本养老和基本医疗保险制度，提高统筹层次。做好城市低保工作。在有条件的地方，建立农村居民最低生活保障制度。加强失业、工伤、生育保险制度建设。积极稳妥地解决农民工和被征地农民的社会保障问题。加快建立城乡特殊困难群众社会救助体系。进一步发展残疾人、社会福利和社会慈善事业。（3）合理调节收入分配。抓紧建立居民收入分配监测和预警体系。规范居民收入分配秩序，调节过高收入，完善国有企事业单位收入分配规则和监管体制。强化个人所得税征管，取缔各种非法收入。（4）积极帮助城乡困难群众解决生产生活问题。加大扶贫开发力度，稳步推进以工代赈和易地扶贫试点。做好灾区群众生活救济、生产恢复和灾后重建工作。完善农村“五保户”供养、特困户救助、灾民救济等制度，增加资金支持并适当提高救助标准。（5）进一步加强安全生产管理。严格落实安全生产责任制，依法关闭取缔不具备安全生产条件的厂矿企业，加大矿山、交通运输、危险化学品、烟花爆竹等重点领域安全生产的治理力度，强化对食品、药品和餐饮卫生等的监管。特别是要按照“企业负责、政府支持”的原则，加大煤矿安全技改和瓦斯治理的投入，企业要提足用好安全生产费用，中央政府要继续安排资金，地方政府也要加大投入，完善通风、防尘、防火、排水、运输等安全生产系统，强化瓦斯抽采和综合利用，坚决遏制重特大事故频发的势头。（6）维护和促进社会稳定。高度重视并认真解决征地拆迁、企业改制、库区移民和环境污染等方面存在的损害群众利益的问题。加快完善社会稳定预警体系和应急处理机制，妥善应对自然灾害、事故灾难、公共卫生以及社会安全等突发事件。健全社会治安防控体系，加强和谐社区、和谐村镇建设，创造城乡居民安居乐业的社会环境。
　　2006年，我国改革发展稳定的任务艰巨而繁重。我们要在以胡锦涛同志为总书记的党中央领导下，高举邓小平理论和“三个代表”重要思想伟大旗帜，全面落实科学发展观，齐心协力，扎实工作，为实现经济社会发展目标和“十一五”的良好开局而努力奋斗！
　　图表一 “十五”期间国内生产总值及其增长率

　　图表二 “十五”期间全国财政收入及其增长情况

　　图表三 “十五”期间粮食产量及增产情况
　　（单位：亿公斤）

　　图表四 “十五”期间规模以上高技术产业增加值情况
　　（单位：亿元）

　　图表五 “十五”期间外贸进出口总额及其增长情况

　　图表六 “十五”期间退耕还林、荒山荒地造林和退牧还草情况
　　（单位：万公顷）

　　图表七　“十五”期间全国义务教育人口覆盖率
　　（单位：%）

　　图表八 “十五”期间城乡居民收入及恩格尔系数变化情况

　　图表九 “十五”期间基本养老保险参保人数及其增长情况

© 北大法宝：（[www.pkulaw.com](https://www.pkulaw.com/)) 专业提供法律信息、法学知识和法律软件领域各类解决方案。北大法宝为您提供丰富的参考资料，正式引用法规条文时请与标准文本核对。
欢迎查看所有[产品和服务](https://www.pkulaw.com/helps/2.html)。法宝快讯：[如何快速找到您需要的检索结果？](https://www.pkulaw.com/helps/70.html)[法宝](https://www.pkulaw.com/helps/69.html)V6有何新特色？

扫描二维码阅读原文

**原文链接：**<https://www.pkulaw.com/chl/e30e3bf38fd8b692bdfb.html>
